# Cardiovascular Profile of Xanthelasma Palpebrarum

**DOI:** 10.1155/2013/932863

**Published:** 2013-06-20

**Authors:** Anupam Dey, Ramesh Aggarwal, Shridhar Dwivedi

**Affiliations:** Hamdard Institute of Medical Sciences and Research, Hamdard University, New Delhi 110062, India

## Abstract

Xanthelasma palpebrarum (XP) are yellow plaques that occur most commonly near the inner canthus of the eyelid and are often associated with atherosclerosis, dyslipidemia, and coronary artery disease. This study was planned to address the issue of associated cardiovascular morbidity in xanthelasma patients attending our cardiac clinic. *Materials and Methods*. A total of 61 patients were detected to be having xanthelasma and constituted the study group. The control group constituted of 130 apparently normal individuals. Each patient underwent detailed history, examination, and investigations. *Results and Discussion*. The most prevalent age group was 40 to 60 years. Males outnumbered females. A percentage of 39.3% of cases had concomitant nicotine addiction. Dyslipidemia was present in 60% of cases, hypertension in 37.7%, prehypertension in 8.77%, diabetes mellitus in 18.03%, and prediabetes in 26.3%. Smokers and obese patients with xanthelasma had a higher prevalence of hypertension. Coronary artery disease (CAD) was found in 6.56% of XP cases. The waist circumference and diastolic blood pressures were significantly higher in XP patients. *Conclusion*. A significant number of cases of xanthelasma palpebrarum are combined with smoking, central obesity, hypertension, diabetes mellitus, and dyslipidemia which are the major risk factors for CAD. Efforts should be made to rule out the same in high-risk xanthelasma subjects.

## 1. Introduction

Xanthelasma palpebrarum (XP) (Greek; *xanthos:* yellow and *elasma: *beaten metal plate) are yellow plaques that occur commonly near the inner canthus of the eyelid, more often on the upper lid [1]. Xanthelasmata can be soft, semisolid, or calcareous and are frequently symmetrical with all four-eyelid involvement. They have a tendency to progress, coalesce, and become permanent. Xanthelasmata represent areas of macrophage-containing lipids, primarily cholesteryl esters, but the exact pathogenesis is not known [2]. Xanthelasmata are composed of xanthoma cells which are foamy histiocytes laden with intracellular fat deposits primarily within the upper reticular dermis. Most studies have found increased concentrations of plasma total cholesterol or low-density lipoprotein cholesterol in people with xanthelasma. It has been known to be associated with atherosclerosis, coronary artery disease, insulin resistance, diabetes mellitus, hypertension, stroke, dyslipidemia, obesity, and hyperuricemia.

Lipid-laden deposits of xanthelasma have been of intense interest among clinicians since long. However, it is still controversial whether such lesions are a marker for cardiovascular or metabolic disease or not. This assumes significance as we are now witnessing comparatively more numbers of xanthelasma subjects in contemporary practice. A study, therefore, was planned to address the issue of associated cardiovascular morbidity in xanthelasma patients attending cardiac clinic at our centre in the period from January 2011 to October 2012.

## 2. Materials and Methods

This study was conducted in the Department of Medicine/Preventive Cardiology at Hamdard Institute of Medical Sciences and Research and HAHC Hospital, New Delhi, India. Patients attending cardiac clinic from January 2011 to October 2012 were screened, and 61 patients were found to have xanthelasma. Informed consent was taken, and they constituted the study group. The control is group constituted of 130 apparently normal individuals who attended the clinic for health checkup as part of the preventive cardiology program. Each patient underwent detailed history and physical examination. Blood samples for hemogram, diabetes, and lipids were collected.

## 3. Results and Discussion

### 3.1. Results

(Table 1) The total number of cases of xanthelasma was 61 while the total number of controls was 130. The youngest case with XP was 21 years while the eldest was 73 years. Prevalence was the highest in the age group of 40 to 60 years (73.77%). Prevalence was higher in males (55.7%) as compared to females (44.3%). Most of the cases (81.97%) were Hindus while 18.03% were Muslims. History of smoking was present in 13 cases (21.3%) and in 21 cases (16.15%) of controls. Eight cases of XP (13.1%) had oral tobacco habit. While xanthelasma was found mostly bilaterally (30 cases; 49.18%), unilateral presentation was found in 15 cases (24.6%). In 10 cases (16.4%), it was present in all four eyelids.

We detected concomitant illnesses in a sizeable number of XP cases. Hypertension was found in 37.7% of cases and 12.17% of controls. Dyslipidemia was found in 60% of cases, diabetes in 18.03%, and CAD in 6.56% of cases. Smokers having XP had higher prevalence of hypertension (43.8%) and CAD (6.2%) as compared to nonsmokers (35.6% and 4.92%, resp.). Likewise, obese patients with XP had a higher prevalence of hypertension (40%) as compared to nonobese ones (12.5%). Nine cases of XP (14.75%) did not have any diabetes, prediabetes, hypertension, prehypertension, dyslipidemia, or obesity.

### 3.2. Discussion

Xanthelasma is fairly prevalent in our population. However, people tend to complain only for aesthetic reasons. Most of our cases were not aware of the significance of these deposits. Age distribution was wide ranging from 21 years to 73 years. We found the peak incidence between 40 and 60 years. This was similar as that reported by Gangopadhya et al. [3] and Jain et al. [2] in their studies from Delhi. They found the majority of patients in the age group of 31–50 years. Most (75.4%) of our cases had multiple (2 or more eyelids) XP. This trend was also found by Ribera et al. [4] and Jain et al. [2] who reported two or more eyelids involvement in 87.9% of the cases.

We found associations with hypertension, dyslipidemia, central obesity, and diabetes in a sizeable percentage of our patients. Jain et al. [2] found that 42.4% of patients had associated systemic diseases like hypertension, CAD, diabetes mellitus, and cholelithiasis.

Dyslipidemia was found in 60% of the 20 cases where serum lipid profile was available. Out of the 49 controls whose lipid profiles were recorded, 57.14% had dyslipidemia. The predominant type of dyslipidemia was hypertriglyceridemia in both cases and controls. Different studies have shown a varying incidence of dyslipidemia in individuals with xanthelasma—ranging from as low as 9.1% to as high as 67.9%. Jain et al. [2] found altered lipid levels in 60.6% of their patients with XP.

CAD was found in 6.6% of cases but none among the controls. Whether or not xanthelasma alone can predict risk of CAD is still not clear, although studies have shown that it can. Christoffersen et al. [5] recently reported that xanthelasma can predict the risk of myocardial infarction, ischaemic heart disease, severe atherosclerosis, and death in the general population, independently of the well-known cardiovascular risk factors.

Pandhi et al. [6] in their study on XP patients found mean carotid intima media thickness (CIMT) significantly higher in XP patients, significant increase in the mean proatherogenic apolipoprotein B, and decrease in the anti-atherogenic apolipoprotein A1 levels in XP patients and mean serum cholesterol, low-density lipoprotein cholesterol, high-density lipoprotein cholesterol, and triglyceride levels similar as in controls. They concluded that XP patients irrespective of their lesions sizes or serum lipid levels should be screened using CIMT for detection of subclinical atherosclerosis.

We found higher prevalence of hypertension in cases of xanthelasma who were smokers as compared to nonsmokers. The fact that 9 cases of XP did not have any comorbid conditions or other cardiovascular risk factors clearly shows that XP can sometimes be a benign occurrence.

## 4. Conclusions

A significant number of cases of xanthelasma palpebrarum are combined with smoking, central obesity, hypertension, diabetes, and dyslipidemia, which are the major risk factors for CAD. A determined effort should be made to rule out prediabetes, prehypertension, and/or dyslipidemia in such high-risk subjects at the earliest opportunity. This will help in preventing future CAD. There is a need for a further study to explore the role of other markers of CAD like CIMT and high-sensitivity C reactive protein (hs-CRP) in high-risk cases of xanthelasma.

## Figures and Tables

**Table 1:** Characteristics of xanthelasma cases and controls.

Variables	Case (*n* = 61)	%	Control (*n* = 130)	%
Age (yrs)				
≤40	6	9.8		
40–60	45	73.77		
>60	10	16.39		
Sex				
Male	34	55.7	77	59.2
Female	27	44.3	53	40.8
Ethnicity				
Hindu	50	81.97	87	66.9
Muslim	11	18.03	38	29.2
Sikh	0	0	5	3.8
Tobacco				
Smoking	13	21.3	21	16.15
Oral Tobacco	8	13.1	6	4.6
Both	3	4.9	5	3.85
Pattern				
Unilateral	15	24.6		
Bilateral (2 eyelids)	30	49.18		
3 eyelids	06	9.8		
4 eyelids	10	16.14		
Associated				
Dyslipidemia	12 (*n* = 20)	60	28 (*n* = 49)	57.14
Conditions				
Hypertension	23 (*n* = 61)	37.7	14 (*n* = 115)	12.17
Diabetes	11 (*n* = 61)	18.03	3 (*n* = 130)	2.3
CAD	04 (*n* = 61)	6.56	0	0
Prehypertension	05 (*n* = 57)	8.77	17 (*n* = 115)	14.78
Prediabetes	05 (*n* = 19)	26.3	12 (*n* = 45)	26.67

XP with smoking: HTN (*n* = 7; 43.8%)				
XP without smoking: HTN (*n* = 16; 35.6%)				

XP with obesity: HTN (*n* = 7; 40%)				
XP without obesity: HTN (*n* = 2; 12.5%)				

XP: xanthelasma palpebrarum; CAD: coronary artery disease; HTN: hypertension.
